# Control in patients with advanced cancer: an interpretative phenomenological study

**DOI:** 10.1186/s12904-022-00984-7

**Published:** 2022-06-01

**Authors:** Andrea Rodríguez-Prat, Denise Pergolizzi, Iris Crespo, Albert Balaguer, Josep Porta-Sales, Cristina Monforte-Royo

**Affiliations:** 1 Faculty of Humanities, Universitat Internacional de Catalunya, Josep Trueta s/n, 08195 Sant Cugat del Vallès, Barcelona, Spain; 2 School of Medicine and Health Sciences, Universitat Internacional de Catalunya, Josep Trueta s/n, 08195 Sant Cugat del Vallès, Barcelona, Spain; 3 Palliative Care Unit, Institut Català d’Oncologia, Hospital Josep Trueta, Girona, Spain; 4 Department of Nursing, School of Medicine and Health Sciences, Universitat Internacional de Catalunya, Josep Trueta s/n, 08195 Sant Cugat del Vallès, Barcelona, Spain

**Keywords:** Advanced cancer patients, Control, Self-efficacy, Qualitative studies, Quality of life, Palliative care

## Abstract

**Background:**

In the context of life-threatening illness, loss of control is argued as a source of suffering and loss of perceived dignity, whereas having control over the dying process has been seen as a way of maintaining personal independence. Little is known about the meaning of control from the patients’ perspectives. Thus, the aim of this study was to explore how patients with advanced cancer understand control, in terms of underlying beliefs, attitudes, and expectations consistent with self-efficacy, in different dimensions of their life, their illness, and their healthcare.

**Methods:**

We conducted semi-structured qualitative interviews using an interpretive phenomenological analysis approach. Patients with advanced cancer from an oncology unit and a palliative care unit from Barcelona (Spain) were recruited. The inclusion criteria were a) ≥ 18 years old; b) fluency in Spanish or Catalan; c) outpatients diagnosed with advanced cancer; d) Eastern Cooperative Oncology Group (ECOG) between 0 and 3; e) judged by their physician or nurse to be emotionally stable; f) considered to have control over their illness and circumstances according to their responsible physician; and g) signed informed consent.

**Results:**

We interviewed eight participants (ages ranged from 29 to 70 years, six were female). Two themes were identified: 1) factors that influence the perception of control, with subthemes: uncertainty about future suffering, character traits underlying a need for control; sense of lack of care as a source of loss of control; and 2) perceiving control over an uncontrollable illness, explained by perceived control over subjective wellbeing and adjusting the focus of control. The data allowed us to identify strategies that promote a sense of control in these patients.

**Conclusions:**

The illness, according to the participants, was experienced as series of losses. However, attention was often focused on areas where they continued to have control. These findings selectively reflect experiences of those who see themselves able to effect outcomes in life, suggesting future research should address how both family members and healthcare professionals can help to empower all patients.

**Supplementary Information:**

The online version contains supplementary material available at 10.1186/s12904-022-00984-7.

## Introduction

In recent years, the commitment to person-centered care as a clinical model has highlighted the importance of empowering patients. As a result, strategies have been designed so that patients are more autonomous and perceive that they have control over their environment, their care and their medical decisions [1].

**Table 1:** Characteristics of the participants and length of the interview

	Sex	Age	Neoplasm	Marital status	Length of the interview
Participant 1	M	70	Lung	Married	1:05:00
Participant 2	F	54	Breast + Metastasis	Divorced	0:59:03
Participant 3	M	65	Lung	Married	0:39:29
Participant 4	F	68	Breast + Metastasis	Single	0:52:38
Participant 5	F	45	Breast	Divorced	0:45:04
Participant 6	F	29	Breast + Metastasis	Partner	0:49:20
Participant 7	F	40	Liver + colon; Metastasis	Partner	1:18:04
Participant 8	F	56	Ovary	Married	0:28:04

**Table 2:** Interview guide

• Some people say that a sense of control, that is having some say over circumstances and being the one to make the decisions, in the context of illness, is important for wellbeing. Do you agree?	
• Some people with your illness think about how their life would be if their illness progresses and they think they would have little control over their life. Have you ever thought about this?	
• Some people usually achieve what they set out to do and have the capacity to cope with unexpected turns of events. Since you became ill you are still as independent as you were before?	
• What aspects of your life do you think you have less control over now? If you have lost control over some aspects of your life, how does this affect you?	
• How do you think you would react if due to your illness you lost control over your life? Say, for example, if you were unable to walk or depended on others…	
• Sometimes wanting to control circumstances is related to a fear of suffering or an uncertain future. Is this true for you or is there something else behind it?	
• Is there anything else you would like to add?	

For patients with advanced illness, concepts of personal control – related to their involvement in care (i.e., autonomy; dependency) and beliefs about their influence over situations (i.e., self-efficacy; locus of control [2]) – are important for multiple reasons. Some studies have shown patients with advanced illnesses voice a desire for control as essential to personal identity and wellbeing [3], feeling needs are met [4], preferences for care and sense of dignity [5, 6]. This may explain why a perceived loss of control has been associated with negative outcomes including distress related to self-perceived burden [7, 8] and the wish to hasten death [6]. Alternatively, maintaining a sense of control has been associated with positive outcomes including psychological adjustment [9], better mental health [10], patient empowerment [11], and treatment decision-making consistent with personal values and goals [12]. Thus, control appears as a multidimensional construct in the literature, relevant to multiple experiences and facets of care for patients with advanced illness.

Although broader understandings of control [13] and autonomy [14] in advanced illness have been reviewed, no qualitative empirical research has directly explored patient understandings of control. In particular, the concept of self-efficacy is defined by the beliefs, attitudes, and expectations derived from experiences in effecting outcomes of life events [2]. This study, therefore, defined patient understandings of control consistent with the theory of self-efficacy – namely, thoughts that explain how people see their influence on event outcomes in the context of advanced illness. The aim of the present study was to explore how patients with advanced cancer understand control, in terms of underlying beliefs, attitudes, and expectations consistent with self-efficacy, in different dimensions of their life, their illness, and their healthcare.

## Methods

### Participants

We recruited advanced cancer patients from the palliative care outpatient clinic of a cancer center and from a general hospital with specialty in oncology and palliative care, both public hospitals, located in the metropolitan area of Barcelona, which primarily serve as a reference for the working middle class population. We included participants from two different hospitals in order to obtain a larger sample for our study.

Inclusion criteria were: a) ≥ 18 years old; b) fluency in Spanish or Catalan; c) outpatients diagnosed with advanced cancer (as defined by the American Society of Clinical Oncology [15]); d) Eastern Cooperative Oncology Group (ECOG) between 0 and 3; e) considered to have control over their illness and circumstances according to their responsible physician (we defined “strong sense of control” by using self-efficacy as a proxy of control). We used the General Self-efficacy scale items in order to define the profile of the eligible participants); f) signed informed consent; and g) judged by their physician or nurse to be emotionally stable to participate in the study. Specifically, they had no symptoms consistent with clinical criteria for mood disorders and could clearly communicate the medical details of their diagnosis, their experiences of the diagnosis and the illness.

Exclusion criteria were: a) an ongoing severe psychiatric disorder, and b) cognitive impairment with score > 5 on the SPMSQ [16]. The project was explained to the palliative care physicians and nurses to purposively select the most suitable informants. Physicians informed the principal investigator (AR) of potential participants. Eleven patients were approached. One patient died before the interview and two patients declined due to poor clinical status. Finally, eight individuals agreed to participate (Table 1).

### Data collection

An interview guide around the key themes was designed (Table 2). We conducted one-off semi-structured interviews with each participant (from January 2016 to March 2018) until data saturation was reached, indicating data, themes and content became repetitive. During the time interviews were carried out, there were no significant changes to treatment and care delivery that would alter a sense of control.

The semi-structured interview examined thoughts of *what* contributed to a maintenance or loss of control, *why* a sense of control was needed in their lives, and *how* a sense of control promoted or interfered with adjustment to illness and dying.

We sought the participants’ preferred moment and location for the interview. The interviews were audio-recorded, transcribed and analyzed. All interviews were performed by the same researcher (AR) with no involvement in these patients’ care to minimize potential bias.

### Data analysis

Data was analyzed using Atlas.ti 8.0 and interpretative phenomenological analysis (IPA), a qualitative approach to understand individual feelings and thoughts pertaining the phenomenon of study [17], ideal to explore individual experiences and understandings of control for those with advanced cancer. We used IPA with the interpretative phenomenology approach so as to focus on interpreting and revealing implicit meanings of their experiences or further understanding each particular individual’s perception of the phenomenon of control.

Audio-recordings were listened to and read multiple times by the principal investigator (AR) and two other investigators (CM, IC).

All transcriptions were read and re-read with the objective of identifying significant statements and the meanings arising from those statements. At first, codes were assigned to each of the meanings that emerged, from a descriptive and textual level. In the readings and analyses following to this initial descriptive reading these codes were classified into categories as a function of conceptual similarity. Finally, codes were grouped into subthemes and themes. The information-rich interviews at the conceptual level helped to analyze and reanalyze the less rich interviews at this level. Although some interviews were brief (one lasted, for example, 28 min), all of them provided valuable information regarding our objective.

The analysis was first carried out in an independent manner by the main researcher (AR). Afterwards, two researchers reanalyzed the data, obtaining similar categories (CM and IC). The preliminary results were shared with all members of the team for discussion until final results were obtained. Member checking of the data within the research team corroborated the interpretation of the data, and no new or further themes were suggested. The study was reported according to the CASP guidelines (Additional material Table 1) [18].

### Ethical approval

All methods were carried out in accordance with the principles of the Declaration of Helsinki. This study was approved by the ethics committees of the two hospitals (Hospital de Bellvitge [PR216/15] and Consorci Sanitari de Terrassa [FR_20171127]). All participants gave written and verbal informed consent. All interviews were anonymized and three researchers had access to transcriptions (AR, CM, IC).

### Personal background

AR is a PhD researcher and holds a BA in Humanities and a Masters in Clinical research. She has conducted several studies with qualitative methods. IC is a PhD and psychologist with expertise in both quantitative and qualitative methods. CM is a PhD and nurse. She has experience in leading research projects combining different methodologies. The different background of all three enriched the analysis of the data. AR brought her philosophical perspective, IC helped in the understanding of the concept of control as a psychological construct, and CM as an important feature of personalized care.

Personal biases were addressed by discussing the emerging categories and disclosing the assumptions of this process of conceptualization.

## Results

Eight participants were included in the study, six female. Ages ranged from 29 to 70 years (Mean = 53.3; Median = 55). All patients were white, five were married or living with a partner. Two patients had lung cancer, four had metastatic breast cancer, one had colon cancer with liver metastasis, and one had ovarian cancer. The interviews lasted from 28 to 78 minutes (Median = 50 minutes).

The analysis identified 240 codes that were grouped into categories classified into 5 subthemes. Finally, two themes were identified: 1) factors that influence the perception of control, and 2) perceiving control over an uncontrollable illness.

### Factors that influence the perception of control

This theme can be understood as a framework through which participants felt that they experienced greater or less control. Three subthemes were identified: 1.1) uncertainty about future suffering, 1.2) character traits underlying the need for control, 1.3) sense of lack of care as a source of loss of control.

These subthemes explain three different ways of a loss or gain of control that were in a sense passive, insomuch as there was no conscious effort to employ control strategies over the circumstances. In this sense, the factors that influence the perception of control can be considered as determined by the illness, by a way of being and by the experience of illness within the healthcare context.

#### Uncertainty about future suffering

Some participants expressed that although the illness had not currently limited them excessively; one of the biggest concerns was uncertainty related to a future loss of independence and control:

“What really [bis] scares me, is not death, death itself, no, because we all have to die, you die and you don’t realize. What scares me is ending up in a wheelchair, having to depend on someone, that, wah - Panic!” (P2)

An unpredictable future loss of physical function, no longer being able to do things, and the anticipation of pain or suffering all threatened their sense of control. Even death was at times less feared compared with the uncertainty of functional deterioration, causing loss of bodily control and independence or suffering:

“Death has usually never scared me, but of course dying... it’s not death... it’s suffering” (P4).

#### Character traits underlying the need for control

Participants explained their character traits that helped them to stay in control, and positioned them to desire or need control. Some individuals explained how personal qualities influenced the way they live and die. Participants talked about their capacity and experiences to overcome difficult situations at different times in their lives and to achieve any objective they had set.

“Yes, this life has taught me a lot. And I have managed to get over anything, from any complicated situation, and I have dealt with things with common sense, as I have always believed … and, okay … my children are proud of me” (P3).

They identified themselves as proactive, planners, independent, and self-sufficient. A common characteristic was how they sought control of their surroundings through organizational skills. The participants explained their precise planning of schedules, diets, medical visits and monitoring of treatment, the use of clinical devices, etc.

“I spent several days sorting things out and I made my list of meals [ …] the day I stop planning, I’ll no longer be me. I am too much of a planner, yes … I usually get things done; I don’t set a goal … unattainable goals, right? [ …] Now with this I’ve had a very hard time, we’ll see how far we can go if not, but I’m doing my bit” (P4).

#### Sense of lack of care as a source of loss of control

##### Misdiagnosis and misinformation

A missed diagnosis at an earlier stage of their illness after visiting different specialists, or the perception that they received inadequate care from their healthcare providers (insufficient information, giving false expectations of the illness, lack of empathy) contributed to a greater perception of a loss of control. The following quotation reflects how some patients received a late diagnosis and, during the process of seeking information about their medical condition, felt they had no control over their situation:

“Everyone said they couldn’t see anything... some of them gave me a bit of medication or some said “that, it’s an inflammation […] but everyone told me that I was senselessly worrying about this” (P4).

##### Negative side effect of the experience of control

In some participants, part of their suffering and experience of lack of control over the situation was generated in the context of the clinical care and as a consequence or side effect of a medical intervention.

Difficult experiences or poor outcomes with treatments, interventions, different medical devices, or experiencing vulnerability due to hospitalization, all conveyed a sense of losing control.

In this quotation, it can be seen how inadequate treatment from clinical staff and an inadequate hospital environment for the patient’s needs contributed to a loss of control:

“[The nurse] put me in an armchair; this is all very painful for me. When I was in that armchair, which was old, my arm hurt … it hurt because I was in a bad position and she left me waiting there for an hour because she made a mistake and put another woman in my chair [ …] And I was waiting and waiting... [I said] “Look, you know what? I have a lot of pain and there is something wrong with this chair”. [Their response] “So, [ …] make a complaint to the [local Government]” (P4).

Another source of a sense of loss of control was the reality of depending on medical devices (e.g. catheterization, colostomy bags, etc.). Those patients described this dependency in terms of “limiting my life” and suffering:

“Everything that wasn’t the treatment, has been the bloody port-a-cath. Pissed off with that... that device here. That was what I felt was limiting my life” (P5).

This experience was even worse when the patients did not know in advance the consequences of the treatment or of the intervention.

### Perceiving control over an uncontrollable illness

The second theme encompassed domains in life that patients’ felt were under their control, beyond their control or both, depending on the circumstances. Patients recognized domains under their control, beyond their control, and a gray area where sometimes they could achieve control, as it related to interactions with others. Circumstances that they took under their control contributed to their subjective wellbeing whereas recognizing circumstances beyond their control contributed to coping strategies. Depending on their perceived control over others, patients could achieve autonomy or expressed a desire to not make others suffer. Two subthemes emerged: 2.1) perceived control over subjective wellbeing, and 2.2) adjusting the focus of control.

#### Perceived control over subjective wellbeing

The interviews revealed the relationship between maintaining control over the situation, being able to make choices about their lives, about treatments and to promote personal wellbeing.

Taking care of themselves through diet and physical exercise, receiving a response from professionals that adapted to their needs, receiving adequate information about medical treatments or having “tied up loose ends with advanced directives”, as commented one participant, contributed to personal wellbeing and maintenance of their sense of control:

“Taking care of your food is a kind of control [ …] because it gives you a feeling that you’re taking care of yourself and that you’re helping to improve your health when you get treatment. Not only through medications, but also that you’re also taking care of your body. Another thing that’s important for me, not just because I have a good time and I enjoy sports. It’s another way to say... feel healthy, all things considered!” (P7).

As long as patients exerted dominance over significant areas of their lives, they felt that “my life continues to be in my hands”, a sentiment expressed by seven participants. The awareness of controlling highlighted that certain realities that were uncontrollable could be distinguished (the fact of having an illness) from those around which they could have a margin of control.

#### Adjusting the focus of control

Participants referenced strategies they had developed to manage their experience of illness, in particular related to strategies that could help them to facilitate the process of acceptation and adaptation within the course of the illness. Deploying these strategies to recognize where they could and could not control their current lives occurred both at a personal and interpersonal level.

##### Personal level

a) Living in the present. The inability to make plans for the future resulted in many attesting that the illness had helped them focus on the present. For some, living 1 day at a time was viewed as a form of control over the only thing really in their hands:

“I plan small things that I’m interested in doing but I don’t plan the future… because then I’d worry now and later and […] it’s not in my power to change the course of events” (P4).

“What are you going to be worrying about? It’s just that, it’s absurd. If you are here today, and tomorrow you don’t know where you are […] Friday they will have the results, and that’s that. But still, at the moment I don’t put myself in that situation, I have to stay more in the now so I don’t lose it if I go. I see it that way” (P5).

Related to living in the present was understanding life as a gift. Going through difficult situations throughout the illness led some participants to value the present and enjoy even temporary wellbeing in contrast with experiences of suffering either from the past or anticipated for the future.

“In the end, you have to view it [life] as a privilege. And basically, if it weren’t for the fact that motivation doesn’t have any privilege, it is. To be able to go to Plugues de la Selva [the forest] from el Montseny [great distances] and take a mind-boggling bike ride and see hermitages and I don’t know where there is no one, because it’s a gift.” (P7).

b) Adapting to the distinct phases of the illness*.* The experience of having overcome challenges of their illness revealed a capacity for adaptation in each moment. One of the patients explained how it seemed they could not stand wearing a colostomy bag, however, when there was no longer an alternative, she was not only able to accept it, but to live as though it was not there. Some patients referred to this as “having surpassed the limits that I didn’t believe I had”:

“You overcome lots of things; if they’d told me everything I’ve been through, and that now I’d be here […] I’d have said that I wouldn’t overcome all of this, I wouldn’t be able to” (P8).

“Yes, yes, that’s it … like a movie. And well, I was seeing myself, my life, I was seeing it as one of those movies. And I said, yikes, but what is happening to me? You know, my hair is falling out! But there comes a time for everything, it’s true eh, one sees that you never get used to it, but you adapt” (P2).

Three participants expressed this idea by highlighting some inconsistency between their expected reactions, and actual actions when the time came: “When the moment comes, you act in a totally different way than what you said previously” (P5).

Another form of channeling the need for control was through the creation of comfort zones, where that person felt most safe and secure. One patient mentioned that away from this comfort zone she felt afraid and insecure so that there was a greater need to remain among surroundings she could control to avoid suffering and distress.

c) Acceptance of what cannot be changed. Living through the illness process meant learning to put life events into perspective and accept (some) circumstances that cannot be controlled:

“I think in the long run I would end up accepting, because that’s what it is; there are people who have struggled with having not even a limb in their body, and have become very strong people. I say, why not me, if I maybe lose just one thing? Because I remember that there is an actor who is a professional swimmer and has no limbs;… if that person manages to do that… I haven’t lost that much either, you know?” (P6).

“You really relativize everything, and when you hear something that makes you say: oh, that’s problematic; you say: well, it will get sorted, if it doesn’t sort itself out one way it will get sorted another way, and if it doesn’t get sorted then it’s not sorted” (P8).

Some individuals were aware they could choose their attitude towards coping; whether negative responses, wondering why they became ill, or focusing on everything they could still do.

All participants indicated there were aspects over which they lost control (or never had control), but was not necessarily experienced as negative, as long as they were able to generate coping strategies without affecting their core values. The patients referenced the progressive deterioration from the illness and activities they could no longer engage in. For those who considered autonomy as a defining aspect of their identity, the thought of not making their own decisions or depending on others was very difficult to cope with and, for one participant, even unacceptable.

“My mother has a very good friend who is a doctor [...] and I’ve told her that if at any time I’m in a bad way [...], I prepare myself with all the pain medication I have, and she’ll make me a good night cocktail and that’s that [...] But they won’t make me suffer and above all … [...] And also, so look, in the end it is the last act of control and I think that’s how it should be. Tell your doctor: ‘Look, don’t worry, okay. So you know, the thing is I can see all your efforts and everything, and it doesn’t motivate me anymore, you know?’” (P7).

##### Interpersonal level

a) Empowering sense of control through the family. Some losses were not experienced negatively, such as the inclusion of loved ones in the care process. Depending on care from others was experienced as a mutual agreement where patients continued to have (albeit limited or diminishing) control. They allowed themselves to be cared for and experienced that help as an extension of their will and control:

“Maybe a time will come when maybe it won’t bother me so much that someone close to me helps me to control things. That won’t bother me” (P4).

Some recognized that others helped them exercise greater control over the situation:

“I believe that my life is still in my hands. The thing is… I depend more on the people around me to achieve my goals […]. But hey, it’s always up to me if I want to accept people’s control over me [...] Obviously I need people to help me, [...] but I’m the one who decides to do one thing or another, and they cannot make me do what others want” (P6).

Also, the family was viewed as an important source of care and help:

“I have had my moments, obviously, where really bad things have happened, but well, I have family where everyone comes together, and they help and… lucky to have them. The family does a lot; my husband, children, I still have parents, siblings. Everyone… I want to say everyone… I want to say… they’ve always been there for me” (P8).

b) Desire not to add to the suffering of others. All participants expressed concern contributing to the suffering of others, perceiving themselves unable to control their impact on or burden to others.

“And it pisses me off that my family, my husband, my kids, or whatever, have to be looking after me, and they have to stop their lives because of me. Let’s face it… if it’s a week, it’s worth a fortnight. But if not… no, I don’t want to be a burden to anyone, and I think everyone around me sees that pretty clearly; except the parents, which is pretty normal. But the rest do” (P8).

Three interviewees experienced caring for a sick relative. This prior experience convinced them they had to protect loved ones from suffering. One patient said she could deal with the illness, but it seemed unfair that her son had to bear any burden also. Another common issue was the inability to control what would happen to their family and loved ones after their death, as participant 1 stated:

“It’s not that I’m worried about dying, as it were. I am more worried about [bis] her [his wife], how she’ll be. Her, my relatives, my friends who care about me, who love me; well... I’m more worried about that than me” (P1).

Another participant mentioned she asked her relatives not to accompany her to the doctor because she knew that without them she was freer to express her emotions.

## Discussion

This study explores patient understandings of control as a multidimensional construct in the context of advanced cancer. Our analysis includes various aspects in the understanding of control: physical (loss of functionality and independence), psychological (coping strategies), and social (relational autonomy)). From this perspective, control can be flexibly defined according to the many meanings to patients influenced by the self – the level of involvement in self-management of their illness or a self-awareness of their responses to distressing events – and by others – the level to which they perceive others support and acknowledge their concerns or are allowed to contribute to their care. This is especially relevant to understanding control under conceptual frameworks such as self-efficacy. Self-efficacy reflects beliefs in a personal ability to effect outcomes in the environment that determine how patients adapt to terminal illness. Although the relationship of self-efficacy to illness has primarily focused on sustained efforts to make lifestyle changes or manage symptoms [19], these findings provide new knowledge of how patients adapt with transient and flexible strategies to approach the challenge of end of life. Here, we expand on how our findings may be applicable to patient self-care, wellbeing, as well clinical practice. The first theme, factors that influence the perception of control, showed the concept of control is context-dependent [20]. The contextual framework marked by the state of the illness, the disposition of the patient and their previous experiences can be considered antecedents that influence the experience of control when living with advanced illness [21].

The uncertainty about future suffering, consistent with previous research [22], was feared and viewed as a threat to control for the majority of patients, marked by anticipating the future impact on physical functioning and further deterioration. Thoughts and fears about the consequences of their illness combined with managing their emotional response is consistent with self-regulation in response to appraisal of health threats [23], which also underlies theoretical response styles to uncertainty [24]. However, these findings offer novel insight beyond what influences uncertainty in illness, but also why it is related to control. Patients here acknowledge that they still plan for their future based on knowledge of their past self. Although this past knowledge informed how they could achieve a sense of control in daily life, it is now challenged by the unknown factors related to their illness that they neither can nor want to plan for. From this perspective, perceived control arises when outcomes can be predicted from the past and any information inconsistent with that past leads to perceived loss of control.

Patients’ interactions with medical staff and treatments contributed to a perceived loss of control. Some authors [25, 26] have stressed that the social and material environment can threat the autonomy of patients. The concept of self-efficacy (often equated to a sense of personal control) reflects perceived control over the self and social environment [27], revealing how interactions influence personal control. Although research on control and autonomy is often focused on the personal perception of patients, our results underscore the importance of the context of care. For example, encouraging a comfortable environment can not only protect from the perception of loss of control but also from loss of dignity [28]. Caring not only relates to treatment but also highlights the importance of effective communication and shared decision making as highly valued aspects of care for patients at the end of their life [29].

Patients also explained their subjective experiences of illness, wellness, and a sense of ‘normality’, despite physical changes, deterioration for decreases in health-related quality of life [30]. When a person evaluates their overall quality of life they are simultaneously assessing their physical, functional, emotional and social state, taking into account their expectations, goals, feelings and personal values. Our findings may highlight the importance of remaining positive, revaluating capabilities and capacity to cope with suffering, and how experiences of disease and related symptoms could be normalized.

The second theme that was identified, perceiving control over an uncontrollable illness, pertained to areas in which patients experienced or desired greater control. Lavoie et al. [3] suggested that exercising autonomy through the accomplishment of smaller daily tasks can generate wellbeing. Participants in the current study relatedly expressed that they could express control by managing their smaller day-to-day meals or medications.

Coping strategies are defined as the set of mechanisms that regulate emotions towards problem-solving [31]. This category was most prominent with a total of 118 quotations grouped into 41 codes. Active and passive acceptance, a positive attitude, focusing on the present were strategies used by participants [32]. Living in the present was among the most used strategies. Just as most of the participants referred to fear of the future, it was also common to pay attention to the present as the only reality they could control [3]. The interpersonal level subtheme conflicts with the ideal of an autonomous and self-sufficient individual. The predominant bioethical model, which holds autonomy as a core value, underscores its more relational over personal significance for human beings. Promoting a culture of relational autonomy, which highlights the essential and positive link from living interdependently, can help to generate narratives that avoid seeing dependence from the standpoint of guilt and burden [3].

There were several methodological challenges we dealt with throughout this study. The fact that none of their participants experienced significant functional limitations was contrary to what we previously considered: the more physical limitations the greater experience of loss of control. However, we found that even these limitations became evident at the time of the interviews; participants were able to subjectively experience a great sense of control. Maybe this can be explained by their strong self-efficacy because one participant, for instance, stated that she felt fine but died some months later. This fact was especially discussed by the research team and contrasted with some physicians involved in the project.

### Contextual representativeness

In the construction of the narratives about meaning-making the personal control, we identify some values and beliefs that are somewhat representative of the cultural background of Spain. On the one hand, some participants claimed to be people with strong religious beliefs that led them to have an accepting attitude towards the disease and, on the other hand, it also became clear that the family is considered an important pillar and an essential element in care [33].

### Implications for practice

The analysis of coping strategies from an interpersonal perspective helped to provide a more contextualized view of control for patients. Advanced illness conveys a vulnerability inconsistent with the model of self-sufficiency, capable of decision-making [20, 34]. Our results suggest including others in decision-making can help avoid the stigma of illness by supporting personal choices in life. However, the desire to not to make others suffer was also reported. Feeling that one is a burden is a source of suffering at the end of their life [35].

The desire for control plays a relevant role in perceived quality of life [36]. Therefore, promoting self-care, improving communication and patient input into treatment decisions, training both patients and family in informed decision making, managing time and living in the present, and favoring conversations about end-of-life concerns can facilitate improved experiences of control [5].

### Limitations

The study is limited to patients with advanced cancer from public hospitals, limiting transferability to populations with other illnesses or socio-economic status. Although the validity of qualitative studies does not depend on simple size, we had only eight participants in the current study. The study participants were predominantly female (6/8); most married (5/8), living in supportive environment and considered to have strong self-efficacy. Results therefore are considered more representative of those with greater social support, and less so for those who perceive less control over their circumstances.

Through data triangulation with both the researchers involved in this study and results published in the literature, we were able to check the consistency of our findings. Nevertheless, due to the difficulty in accessing our population and gatekeeping among professionals to protect patients from situations that can further emotional distress, it was a challenge to obtain a greater number of participants in our sample. The difficulties in gaining an appropriate number of participants explain the length of time for the fieldwork.

## Conclusions

This study conceptualizes patient understandings and experiences of control as a multidimensional construct in the context of advanced cancer. Although the illness was experienced as a set of losses, participants focused on areas where control could still be maintained. These results emphasize that family and healthcare professionals can contribute to empower patients.

## Supplementary Information

**Additional file 1.**

## Data Availability

Due to privacy and ethical concerns, neither the data nor the source of the data can be made available. Those interested can contact the corresponding author for more information.
