# Supplementary material for: Control in patients with advanced cancer: an interpretative phenomenological study
Source: BMC Palliat Care. 2022 Jun 1;21:97. doi: 10.1186/s12904-022-00984-7 (PMC9161471; doi:10.1186/s12904-022-00984-7)
Supplement: Supplementary file 1 — Additional file 1. [file 12904_2022_984_MOESM1_ESM.doc]

| **CASP: Critical Appraisal Skills Programme** |  |
| --- | --- |
| – What the goal of the research was | ✓ |
| – Why it is important? | ✓ |
| – Its relevance? | ✓ |
| – If the research seeks to interpret or illuminate the actions and/or subjective experiences of research participants | ✓ |
| – If the researcher has justified the research design (e.g. have they discussed how they decided which methods to use?) | ✓ |
| – If the researcher has explained how the participants were selected | ✓ |
| – If they explained why the participants they selected were the most appropriate to provide access to the type of knowledge sought by the study | ✓ |
| – If there are any discussions around recruitment (e.g. why some people chose not to take part) | ✓ |
| – If the setting for data collection was justified | ✓ |
| – If it is clear how data were collected (e.g. focus group, semi-structured interview, etc.) | ✓ |
| – If the researcher has justified the methods chosen | ✓ |
| – If the researcher has made the methods explicit (e.g. for interview method, is there an indication of how interviews were conducted, did they used a topic guide?) | ✓ |
| – If methods were modified during the study. If so, has the researcher explained how and why? | -- |
| – If the form of data is clear (e.g. tape recordings, video material, notes, etc.) | ✓ |
| – If the researcher has discussed saturation of data. | ✓ |
| – Formulation of research questions | ✓ |
| – Data collection, including sample recruitment and choice of location | ✓ |
| – How the researcher responded to events during the study and whether they considered the implications of any changes in the research design | -- |
| – If there are sufficient details of how the research was explained to participants for the reader to assess whether ethical standards were maintained | ✓ |
| – If the researcher has discussed issues raised by the study (e. g. issues around informed consent or confidentiality or how they have handled the effects of the study on the participants during and after the study) | ✓ |
| – If approval has been sought from the ethics committee | ✓ |
| – If there is an in-depth description of the analysis process | ✓ |
| – If thematic analysis is used. If so, is it clear how the categories/themes were derived from the data? | ✓ |
| – Whether the researcher explains how the data presented were selected from the original sample to demonstrate the analysis process | ✓ |
| – If sufficient data are presented to support the findings | ✓ |
| – To what extent contradictory data are taken into account | ✓ |
| – Whether the researcher critically examined their own role, potential bias and influence during analysis and selection of data for presentation | ✓ |
| – If the findings are explicit | ✓ |
| – If there is adequate discussion of the evidence both for and against the researcher’s arguments | ✓ |
| – If the researcher has discussed the credibility of their findings (e.g. triangulation, respondent validation, more than one analyst.) | ✓ |
| – If the findings are discussed in relation to the original research questions | ✓ |
| – If the researcher discusses the contribution the study makes to existing knowledge or understanding (e.g. do they consider the findings in relation to current practice or policy, or relevant research based literature?) | ✓ |
| – If they identify new areas where research is necessary | ✓ |
| – If the researchers have discussed whether or how the findings can be transferred to other populations or considered other ways the research may be used | -- |

**Supplementary table.** Methodological Quality of included studies assessed with CASP: qualitative research checklist.
